# Osteitis pubis: can early return to elite competition be contemplated?

**Published:** 2014-04-08

**Authors:** Jaume Jardí, Gil Rodas, Carles Pedret, Lluis Til, Manuel Cusí, Nikolaos Malliaropoulos, Angelo Del Buono, Nicola Maffulli

**Affiliations:** (1) F.C. Barcelona Medical Department. Barcelona. Spain; (2) Centre de Diagnòstic per Imatge de Tarragona. Tarragona. Spain; (3) Sydney School of Medicine. University of Notre Dame. Sydney. Australia; (4) National Track & Field Centre, Sports Medicine Clinic of S.E.G.A.S., Thessaloniki, Greece; (5) Department of Orthopaedic and Trauma Surgery, Campus Biomedico University of Rome, Via Alvaro del Portillo, Rome, Italy; (6) Centre Lead and Professor of Sports and Exercise Medicine Consultant Trauma and Orthopaedic Surgeon Centre for Sports and Exercise Medicine Barts and The London School of Medicine and Dentistry Mile End Hospital

**Keywords:** Osteitis pubis, Rehabilitation, Lumbo-pelvic stabilization, sport

## Abstract

**Background and purpose:**

In elite athletes, osteitis pubis is a common painful degenerative process of the pubic symphysis and surrounding soft tissues and tendons. We report the diagnostic pathway and the rehabilitation protocol of six elite athletes with osteitis pubis in three different sports, and compare protocol stages and time to return to competition.

**Methods:**

6 athletes (2 soccer, 2 basketball, 2 rugby players) were diagnosed with osteitis pubis stage III and IV according to Rodriguez classification using standard clinical and imaging criteria. After performing a baseline lumbo-pelvic assessment, the rehabilitation protocol described by Verrall was adapted to each individual athlete.

**Results:**

The length of time for each stage of the protocol was as follows; Stage 1 (rest from sport) was 26 +/− 5 days, Stage 2 (to achieve pain free running), 18 +/− 5 days, Stage 3 (squad training) 63 +/− 7, Stage 4 (return to competition) 86 +/− 15. Soccer players took longer to return to competition than basketball and rugby players. No recurrences were reported at 2 year follow-up.

**Conclusion:**

The protocol presented ensures a safe return to elite athletes. The time from diagnosis to full recovery is longer in football players, and seems to increase with age.

## Introduction

Osteitis pubis, a common cause of groin pain in elite athletes, especially kicking sports^1^, is a degenerative process which involves the pubic symphysis and surrounding soft tissues and tendons. The changes at the symphysis may be asymptomatic, but athletes often present debilitating symptoms, slow recover, and high recurrence rate^2^. The pathogenesis is still debated, the terminology unclear, and many causes, including “sports hernia”^3^, “athletic hernia”^4^,^5^, “osteitis pubis”^6^,^7^,^8^,^9^, “athletic pubalgia”^10^, and “adductor-related groin pain”^11^, and “dynamic pubic osteopathy” may have to be taken into account as differential diagnose. The new concepts of “sports-related chronic groin injury” and ‘groin disruption injury’ describe a condition of chronic groin pain associated with pubic instability^12^, an overuse syndrome often observed in footballers, ice hockey and tennis players, and, occasionally, in non-athletes, in whom the pelvic instability may occur as consequence of post-partum diastasis of the symphysis or traumatic pelvic diastasis. However, very common in track and field athletes practicing kicking sports such as soccer, gridiron, Australian rules football, rugby and martial arts such as Tae-kwon-do, osteitis pubis accounts for 0.7 to 7% of all sport related injuries, with a high incidence in soccer players (5–13%)^7^,^12^,^13^. Multi-factorial in its etiology, even though it is often misdiagnosed and chronic, early diagnosis and a multilateral therapeutic approach are mandatory. The diagnosis is usually clinical, based on clinical history, physical examination, and functional assessment^7^,^8^,^12^. Imaging is advocated to exclude adductor, abdominal, and hip pathologies, nerve entrapment, and several forms of unspecified referred pain which often occur simultaneously, or simply act as predisposing factors. First line management is conservative, and surgery is indicated in unresponsive patients^4^,^14^,^15^,^7^. Prevention programs based on specific sport related demands should be tailored to the needs of each individual athlete^16^,^17^,^18^. Long rehabilitation is often needed^1^, with recovery times varying from 2 to 12 months^1^. Osteitis pubis has a negative impact on the career of an athlete, who may be obliged to stop their sporting activities. In 5–10% of these patients, surgery may be necessary^19^.

The present study reports the duration of each rehabilitative stage, and the rehabilitation interval (time from diagnosis to return to competition) of six elite athletes (2 basketball players, 2 soccer players, and 2 rugby players) undergoing a standardized rehabilitation protocol for management of osteitis pubis.

## Materials and Methods

From 2003 to 2006, 6 elite athletes with clinical suspicion for osteitis pubis were assessed at the Centre for sports and exercise medicine “Consorci Sanitari del Garraf” (Barcelona, Spain). Inclusion criteria for diagnosis of osteitis pubis were unilateral or bilateral groin pain, soft symphysis, tender to palpation, positive squeeze test, evidence of bone marrow oedema on MRI scans, and increased uptake to the pubic bone on Tc99 triple phase bone scan^12^.

MRI findings were graded according to subchondral bone oedema, fluid in the pubic symphysis, and periarticular oedema^6^. The criteria defined by Cunningham et al^20^ and Zoga et al^3^ (image 1) were also used. Bone scans were graded according to Balius^21^.

Anthropometric data were collected (Table 1). At clinical assessment, all patients were staged as 3 or 4 according to Rodriguez et al^8^ (Table 2).

The Wisbey-Roth Core Stability Grading System was used to classify lumbo-pelvic stability, and ascertain whether osteitis pubis had any effects on lumbo-pelvic stability (Table 3). The patients’ core stability grading is summarized in Table 4.

## Rehabilitation Protocol

Wollin’s protocol^22^ was used as baseline, and Verrall’s indications^12^ were followed, with some modification. Athletes were moved through the protocol stages after they had been able to perform correctly exercises with no pain, and had achieved adequate levels of movement and core stability grading. The protocol consists of 3 stages.

**Stage I (pain control)** In this stage, patients rest for 3–4 weeks, and undergo RICE, and alternate cold (12º–14º) and hot water (31º) baths. Controlled exercises in a swimming pool are allowed, together with deep muscle massages. Static isometric strength exercises of the pelvic floor, of the transversus abdominis, multifidus and diaphragm, and of the adductors, and abdominal wall muscles are also started, allowing only light concentric exercises of these muscle groups, avoiding high intensity or high volume workouts. Gentle prolonged stretching for all above muscle groups, except for the adductors and ischio-pubic muscles, were also allowed. Cycling on an exercise bike, introduced as cardiovascular training, is initially allowed for 10 minutes per day, and gradually increased. As end stage goal, athletes have to achieve Grade 2 Wisbey-Roth lumbo-pelvic stability.

**Stage II** Swiss balls and other aids are indicated to perform resistance and strength contraction exercises of the pelvic floor, transversus abdominis, and multifidus, muscles, and to vary amplitude and rhythm of contraction. Gluteal strengthening is started, and cycling on an exercise bike is allowed for 20 min per day. Changes of speed and resistance are introduced, and dynamic strengthening of the muscles around the hip in flexion, extension, adduction, and abduction are permitted by using an elastic band. The primary objective is to achieve a Grade 3 Wisbey-Roth lumbo-pelvic stability. In the absence of symptoms, patients start with a maximum 30 minutes program alternating fast walk, running, and walking.

**Stage III** Eccentric work on the sliding board is started, initially with a 1 metre lateral slide (3 sets, 30 seconds each), progressively increased in terms of slide distance and number of sets. Running time is gradually increased, and changes of pace and direction are introduced. To reproduce the sport requirements, athletes start training on the field or using indoors circuits, performing exercises mimicking their sport. Kicking is allowed only at the end of this stage. Eccentric abdominal wall strengthening exercises are started. Athletes aim to achieve grade 4 Wisbey-Roth lumbo-pelvic stability. At this stage, athletes start progressive squad training. At this stage, they can progressively practice sport, up to play in competition matches.

**Return to play (competition) criteria** Factors such as type of sport, level of competition, and personality of the athlete, contribute to return to training and competition. The criteria followed are outlined in Table 5. Clinical assessment and level of lumbo-pelvic stability helped to decide when patients could be discharged and could return to sport.

## Results

The duration of the different stages is reported in Table 6 for all six athletes. Timing of rest from sport activity varied from 21 to 35 days. Rugby players started running earlier than the other athletes, after an average period of 13.5 days from the beginning of the rehabilitation program. The 2 basketball players started squad training earlier than the others, 48 and 60 days after they had started rehabilitation. Basketball players were the first to return to sport activity, 57 and 88 days after they had started rehabilitation. Even though the time from the onset of symptoms to diagnosis had been considerably delayed, almost all athletes (5 of 6) returned to competition in less than three months.

## Discussion

The main finding of the present study is that it is possible to return to elite competition without recurrence when applying rehabilitation protocols tailored individually to each athlete. In our small cohort of elite athletes, the rehabilitation interval (time from diagnosis to discharge) was shorter in basketball and rugby players, and younger players recovered earlier than older ones. Strict diagnostic criteria, treatment protocols, and rehabilitative measures tailored to the individual needs are therefore suggested. Although the present study reports on only 6 elite athletes, all were competing in high level leagues. We tried to apply strict diagnostic criteria and rehabilitation protocols in all the patients to ensure comparable assessments and results.

Lumbo-pelvic stability (“core stability”)^23^ was graded as it is involved in OP, and may be useful to establish standard rehabilitation protocols for these patients. We applied the tensegrity model^24^ to manage the process. Poor neuromuscular control on trunk and pelvis can be a risk factor for sports injury, and poor abdominal strength or endurance may predispose to recurrent injuries to hamstring^25^, lower back and knee. Conversely, osteitis pubis can be easily triggered in subjects with adductor tightness. We used the grading system proposed by Wisbey-Roth to assess the severity level of the condition, and stage the rehabilitation protocol. We did not use ultrasound nor neoprene shorts to control groin pain^26^.

Few studies have compared different rehabilitation protocols for management of OP, and available outcomes are not comparable with the present work. It is difficult to establish evidence-base criteria to determine *a priori* the length of time away from sport and interval time of progressive stages of rehabilitation. However, most authors suggest an initial resting period from 2–4 weeks to 5–6 months, according to the severity of symptoms and grade of lumbo-pelvic stability. Usually, accepted intervals vary from 4 to 6 weeks for stage I, from 6 to 8 weeks for stage II, from 9 to 12 weeks for stage III, and from 4 to 5 months for stage IV^25^,^8^,^22^. In the present study, all the athletes were stages III and IV, and 5 of 6 returned to sport within three months (10–13 weeks), a reasonably shorter time period compared to other studies^8^.

Different outcomes were reported in relation to sport activity, but the small number of enrolled subjects precludes statistical analysis. From a clinical perspective (history and physical examination), it is reasonable to conclude that soccer players had more symptoms related to the “round the corner” ball kicking action. The duration of time off sport depends on clinical, imaging, and lumbo-pelvic stability related findings. The rest period ceases when the squeeze test is not painful. After rest, generally calisthenics, stationary biking and treadmill walking were allowed for an average of 2 weeks before continuous running was permitted. The patients took an average time of 2 months to start squad training, and 3 months to return to competition. Basketball players had the shortest recover, followed by rugby and football players. Follow-up after at least 2 seasons showed no recurrences.

This study presents a group of elite athletes who successfully returned to their previous high level competition following treatment of osteitis pubis (Table 7). Weaknesses of the study are the small number of patients, the absence of a control group. We are also aware that the Wisbey-Roth Grading System has a fair to poor inter-tester reliability 27.

## Conclusions

Osteitis pubis is a functional pathology related to altered neuromuscular control of the pelvis. Initially well tolerated, it may progress to a chronic state, can be debilitating, and result in prematurely interruption of sporting careers. Clinical and imaging criteria are available to classify the evolution of the chronic stage of osteitis pubis. The assessment of lumbo-pelvic stability may be helpful to assess the timing and monitoring the stages of the rehabilitation program, allowing an early return to competition.

## Figures and Tables

**Table 1.** Anthropometric and sport data

**Sport**	**Patient 1 Soccer**	**Patient 2 Soccer**	**Patient 3 Basketball**	**Patient 4 Basketball**	**Patient 5 Rugby**	**Patient 6 Rugby**
Age (years)	21	30	30	27	21	17
Height (cm)	170	180	192	206	185	186
Weight (Kg)	64	70	90	93	86	83
Field position	Midfielder	Forward	Base	Centre	Centre	Flanker
Dominance	Right	Right	Right	Right	Right	Right

**Table 2.** Stages of osteitis pubis

	**Side of pain**	**Site of pain**	**Characteristics of pain**
**Stage 1**	Unilateral, dominant	Inguinal, with radiation to adductors	Mechanical, settles after warmu, returns after training,
**Stage 2**	Bilateral	Inguinal and adductors	Increases after training.
**Stage 3**	Bilateral	Groin, adductor region, suprapubic, abdominal	During training, kicking, sprinting, turning. Cannot achieve training goals, forced to withdraw
**Stage 4**	Generalised	Generalised, radiation to lumbar region	Walking, getting up, straining at stool, simple activities of daily living

**Table 3.** Wisbey-Roth Core Stability Grading System

**Grade 0**	Unable to maintain an isometric contraction without compensatory movement of the core (i.e. lumbo- sacral and pelvis) in a position aimed to facilitate the stabilising role of key muscles.
**Grade 1**	Able to maintain an isometric contraction (10 to 20 seconds) without compensatory movement of the core (i.e. lumbo-sacral and pelvis) in a position aimed to facilitate the stabilising role of key muscles.
**Grade 2**	Able to maintain an isometric contraction (for 20 seconds) without compensatory movement of the core with superimposed slow movement of the limbs.
**Grade 3**	Able to maintain an isometric contraction (for 20 seconds) without inappropriate movement of the core while performing slow movements of the trunk itself.
**Grade 4**	Able to maintain an isometric contraction (for at least 20 seconds) without compensation /inappropriate movement of the core while performing fast movements of the limbs.
**Grade 5**	Able to maintain an isometric contraction (for at least 20 seconds) without compensation/inappropriate movement of the core while performing one of the following: - Fast movements of the trunk - if appropriate to activity required, with joint angle specific positioning and muscle function specific. Fast movements of the limbs in joint angle specific postures and muscle function specific (i.e. reproducing concentric/eccentric role of key stabilisers) Against increased resistance/increased load in joint angle specific postures and muscle function specific positioning, which are sport/activity specific.

**Table 4.** Core stability grading of the six athletes

Sport	Patient 1 Soccer	Patient 2 Soccer	Patient 3 Basketball	Patient 4 Basketball	Patient 5 Rugby	Patient 6 Rugby
Lumbo-pelvic core stability grading	2	1	1	2	1	2

**Table 5.** Return to play criteria

***Clinical guidelines***	Symptom free for 1 month minimum (negative squeeze test) Pain free palpation of symphysis and pubic rami. Pain free stretching, isometric (concentric and eccentric) contraction of adductors Negative osteopathic assessment, both structural, functional and viscerally. Manages well all the strengthening exercises without pain, lumbo- pelvic stability = Level 4 Able to do concentric-eccentric work of rectus abdominis and both obliques (2 sets of 7 repetitions, three times a week)

***Fitness criteria***	Achieves pre-injury times and intensities (i.e. beep test) or running for 20 minutes at three different intensities). Completed full squad training sessions without any problems (2 weeks for stages 3 and 4) Completed a full game (friendly, lower grades) without symptoms

**Table 6.** Average duration of rehabilitation stages (individual values in brackets)

	**Football**	**Basketball**	**Rugby**	**Overall average**
**Rest from sport (days)**	28 (21–35)	24.5 (28–21)	24.5 (28–21)	25.7
**Start of running**	21 (14–28)	21 (21–21)	13.5 (17–10)	18.5
**Start of squad training**	80.5 (58–103)	54 (60–48)	57.5 (78–43)	63
**Return to competition**	96.5 (72–121)	72.5 (88–57)	89.5 (105–74)	86.2

**Table 7.** Clinical progress of each athlete

Sport (Dominance)	Patient 1 Soccer (Right)	Patient 2 Soccer (Right)	Patient 3 Basketball (Right)	Patient 4 Basketball (Right)	Patient 5 Rugby (Right)	Patient 6 Rugby (Right)
OP Stage	III	IV	IV	III	III	IV
Site of initial symptoms	Pubic symphysis	Right Adductor longus	Bilateral adductor insertion	Right adductor longus	Right adductors	Adductor longus and symphysis
Development of symptoms	Suprapubic, Right Oblique	Infrapubic Right adductor	Acute low back pain	Pubic symphysis	Pubic symphysis	Suprapubic, bilateral
Lumbo-pelvic stability	Level 2	Level 1	Level 1	Level 2	Level 2	Level 1
Diagnostic delay	6 months	8 months	4 months	4 months	8 months	24 months
Relative rest	21 days	35	82	21	28	21
Start of running	14	28	21	21	21	10
Time to squad training	58	103	60	48	35	43
Return to play	72	211	88	67	91	74
Symptom free follow up	48 months	36 months	30 months	24 months	24 months	18 months
